# Immunogenomics and spatial proteomic mapping highlight distinct neuro-immune architectures in melanoma vs. non-melanoma-derived brain metastasis

**DOI:** 10.1038/s44276-024-00060-y

**Published:** 2024-05-02

**Authors:** Alberto Mendoza-Valderrey, Ethan Dettmann, Douglas Hanes, Daria M. Kessler, Ludmila Danilova, Kai Rau, Yueqin Quan, Stacey Stern, Garni Barkhoudarian, Carlo Bifulco, Kim Margolin, Steven Kolker, Maria L. Ascierto

**Affiliations:** 1 Translational Cancer Immunology and Immunotherapy Department, Saint John’s Cancer Institute, Providence Saint John’s Health Center, Santa Monica, CA USA; 2 Providence Research Network, Portland, OR USA; 3 Bioinformatic Department, Saint John’s Cancer Institute, Providence Saint John’s Health Center, Santa Monica, CA USA; 4 Division of Quantitative Science, Department of Oncology, School of Medicine at Johns Hopkins, Baltimore, MD USA; 5 Biostatistics Department, Saint John’s Cancer Institute, Providence Saint John’s Health Center, Santa Monica, CA USA; 6 Neurosurgery Division, Pacific Neuroscience Institute, Santa Monica, CA USA; 7 Earle A. Chiles Research Institute, Genomic and Pathology Department, Portland, OR USA; 8 Melanoma Program, Saint John’s Cancer Institute, Providence Saint John’s Health Center, Santa Monica, CA USA; 9 Dermatopathology Section, Providence Saint John’s Health Center, Santa Monica, CA USA

## Abstract

**Background:**

Brain metastases (BrMs) are a devastating complication of solid tumours. A better understanding of BrMs biology is needed to address their challenging clinical management.

**Methods:**

Immunogenomic and digital spatial analyses were applied to interrogate the peripheral blood and tumour specimens derived from 53 unique patients with BrMs originating from different solid tumours.

**Results:**

At craniotomy time, patients with melanoma-derived brain metastasis (MBM) displayed in the periphery lower neutrophil–lymphocyte ratio (NLR) compared to non-melanoma-derived brain metastasis (non-MBM). Regardless of the primary tumour source, higher NLR was associated with reduced overall survival (OS). Tumour MicroEnviroment genomic evaluations revealed higher expression of genes identifying NK, CD8 and B cells in MBM vs. non-MBM. Moreover, MBM patients with longer OS displayed increased CD8+ cell infiltration. Spatial proteomic analysis further highlighted enriched infiltration of CD8+ cells, antigen-presenting cells, T-cell agonists and B cells in MBM. Conversely, increased expression of genes and proteins associated with neurodevelopment, cell–cell adhesion and neutrophil infiltration were observed in non-MBM.

**Conclusions:**

These findings reveal an increased immunogenicity of MBM vs non-MBM and highlight the presence of a unique neuro-immune interplays in MBM vs non-MBM, suggesting that a balance between neuro-immune architectures might be associated with diverging clinical outcome of patients with BrMs.

## Introduction

Brain metastases (BrMs) are the most frequent central nervous system (CNS) malignancy and represent a fatal cancer complication with unmet therapeutic needs [1]. In the United States, the estimated number of BrMs cases diagnosed each year is between 98,000 and 170,000 [2], with melanoma, breast and lung cancers being the most common histologies (incidence of disease and percent developing brain metastases contributing to their relative numbers and the overall total) [3]. Among them, melanoma has the highest frequency of metastases to the brain [4]. The standard of care therapies for BrMs management, including surgical resection and/or radiotherapy, have historically resulted in a difficult control of tumour progression reporting poor survival rates [5]. The difficult clinical management of BrMs is mainly due to the fact that the resident cells within the CNS create a complex and dynamic microenvironment, with interactions among neurons, astrocytes, oligodendrocytes, microglia, immune cells, and extracellular matrix, all essential to normal function [6, 7]. This ecosystem is separated from the peripheral vasculature by the blood-brain barrier (BBB), a selective filter composed of tightly connected endothelial cells, pericytes, and astrocyte projections within a dense basement membrane. The invasion of metastatic cells into this ecosystem results in an evolving TME distinct from any other seen in systemic metastasis [1].

Recent studies have demonstrated that immune checkpoint blockade (ICB) have efficacy in the treatment of BrMs derived from melanoma and non-small cell lung cancer (NSCLC), improving the survival outcomes in these patients [8–10]. Particularly, the phase 2 CheckMate 209-204 trial evaluated the efficacy of monoclonal antibodies blocking the Cytotoxic T-Lymphocyte Antigen 4 (CTLA-4) and the Programmed Death 1 Receptor Pathway [PD-(L)1] in 94 patients with melanoma brain metastasis (MBM) and reported an intracranial and similar extracranial/global response rate of 55%, with a partial response rate of 30% and complete response rate of 26%. In breast cancer, ICB targeting PD-L1 has been approved in combination with nab-paclitaxel for the treatment of triple-negative metastatic breast cancer (MBC) [11]. As in melanoma, if immunotherapy proves effective for MBC, patients with breast-derived brain metastasis (BBM) could also benefit from an intracerebral response. This hypothesis is leading to the development of novel immune-oncology clinical trials for the treatment of patients with BBM.

The rapid development of novel immune-oncology clinical trials able to address the challenging clinical management of patients with BrMs leads to an urgent need to better explore the immune portrait of BrMs derived from different solid tumours in order to better inform the development of novel and efficacious treatment strategies [12, 13]. However, to our knowledge, no study has comprehensively examined the immune landscape of the tumour microenvironment of derived BrMs from different solid tumours. This is critical to uncovering more immune-responsive BrMs as compared with more immune-resistant ones. To address this gap in knowledge, we analysed a set of surgically resected BrMs originating from different solid tumours, including melanoma, lung cancer, breast cancer and renal cell carcinoma (RCC), using whole gene expression profiling, digital spatial proteomics (DSP), immunohistochemistry (IHC), multiplex immunofluorescence (mIF) and spectral flow cytometry.

Although multiple studies clearly reported decreased immunogenicity of BrMs when compared to extracranial metastases (ECM) and primary tumours [14–16], we hypothesised that the TME of MBM is more immunogenic when compared to BrMs derived from other solid tumours (non-MBM). The results of our integrated and multidimensional comparative study may help guide the development of novel patient selection strategies and novel therapeutic strategies for improving the clinical outcome of patients with brain metastases.

## Methods

Detailed information is available in Supplementary Materials and Methods.

### Patient cohort and specimens

Samples derived from 59 unique patients (53 with BrMs and 6 with Primary Melanoma) treated at the Saint John’s Health Center (Santa Monica, CA) between 2004 and 2023 were evaluated in this study, which was approved by the Providence Health Center Institutional Review Board (IRB). Patients consented to the collection of blood and/or tumour specimens for research. Detailed information for the patient cohort evaluated in this study is shown in Table 1, Supplementary Table 1 and Supplementary Materials and Methods.

**Table 1:** Demographics and type of treatments received across the different BrMs types.

	Melanoma-derived BrM (MBM) (*N* = 14)	Lung cancer-derived BrM (LBM) (*N* = 16)	Breast cancer-derived BrM (BBM) (*N* = 18)	RCC-derived BrM (RBM) (*N* = 5)
Mean age at diagnosis of BrMs (years)	67.6	66.8	55.9	68.8
Gender patients (% out of BrMs type)				
Female	5 (35.7%)	8 (50%)	18 (100%)	1 (20%)
Male	9 (64.3%)	8 (50%)	0 (0%)	4 (80%)
Treatment received before BrMs diagnosis (% out of BrMs type)				
Surgery	13 (92.9%)	6 (37.5%)	15 (83.3%)	1 (20%)
Chemotherapy	1 (7.1%)	4 (25%)	17 (94.4%)	0 (0%)
Radiotherapy	2 (14.3%)	3 (18.8%)	10 (55.6%)	1 (20%)
Immunotherapy	5 (35.7%)	3* (18.8%)	0 (0%)	0 (0%)
Others (i.e., HER2 targeted therapy)	0 (0%)	1 (6.3%)	16 (88.9%)	2 (40%)
Untreated	0 (0%)	6 (37.5%)	0 (0%)	1 (20%)
Unknown	0 (0%)	1 (6.3%)	0 (0%)	2 (40%)
Treatment received after BrMs diagnosis (% out of BrMs type)				
Surgery	12 (85.7%)	16 (100%)	18 (100%)	5 (100%)
Chemotherapy	4 (28.6%)	2 (12.5%)	10 (55.6%)	1 (20%)
Radiotherapy	11 (78.6%)	14 (87.5%)	18 (100%)	3 (60%)
Immunotherapy	6 (42.9%)	7 (43.8%)	3 (16.7%)	2 (40%)
Others (i.e., HER2 targeted therapy)	3 (21.4%)	2 (12.5%)	14 (77.8%)	3 (60%)

*Two of the three LBM patients receiving IO before the diagnosis of brain metastases had two BrMs FFPE samples collected at the time of craniotomy, leading to a final number of 5 FFPE specimens derived from three unique LBM patients assessed by RNAseq and IHC. Information regarding specimens used for the different assessments conducted in this study is provided in Table 2.

### Gene expression profiling and analysis

Tumoral regions were carefully identified by the pathologist and were manually dissected from 5-μm tissue Formalin-fixed, paraffin-embedded (FFPE) sections as previously described [17]. Total RNA was isolated from selected tumour areas with the High Pure RNA Paraffin Kit (Roche Diagnostics GmbH, Mannheim, Germany) following the manufacturer’s guidelines. Global gene expression profiling was assessed by Illumina and further analysed in a total of 54 FFPE specimens derived from 50 unique patients with brain metastasis. More information regarding the FFPE specimens used for gene expression profiling in this study are provided in Table 2. The EdgeR [18] package with a negative binomial model and common dispersion estimates was used to calculate the Differentially Expressed Genes (DEG) between the groups of interest. DEG were identified based on a significant *P* value (adjusted *P* value ≤ 0.05). Functional analysis was performed using the Ingenuity Pathway Analysis (IPA) software. Only significant pathways derived from IPA analysis were reported in this study.

**Table 2:** List of the specimens derived from primary tumours and BrMs evaluated in the study.

Patient #	PT FFPE sample ID	BrMs FFPE sample ID	Circulating immune cells information	TIL sample ID
1	NA	MELBRM1-B^a,c,d^	NA	NA
2^$,&^	MELBRM2-A^b,c^	MELBRM2-B^a,b,c^	X	NA
3^$^	NA	MELBRM3-B^a,c^	NA	NA
4^&^	NA	MELBRM4-B^a,c^	X	NA
5^$^	NA	MELBRM5-B^a,c,d^	NA	NA
6^$^	NA	MELBRM6-B^a,c^	X	NA
7^$^	NA	MELBRM7-B^a,c^	NA	NA
8	MELBRM4-A^b,c^	MELBRM8-B^a,b,c^	NA	NA
9^&^	NA	MELBRM9-B^a,c,d^	X	NA
10^&^	NA	MELBRM10-B^a,c^	X	NA
11	NA	MELBRM11-B^a,c,d^	X	NA
12^&^	NA	MELBRM12-B^a,c,d^	X	NA
13^&^	NA	MELBRM13-B^a,c,d^	NA	NA
14	NA	NA	NA	MBM-TIL-14
15	NA	NA	NA	SKCM-TIL-15
16	NA	BREBRM19-B^a,b,c^	NA	NA
17	NA	BREBRM20-B^a,b,c^	X	NA
18	NA	BREBRM21-B^a,c,d^	X	NA
19	NA	BREBRM23-B^a,c^	X	NA
20	NA	BREBRM24-B^a,c^	NA	NA
21	NA	BREBRM25-B^a,c,d^	NA	NA
22	NA	BREBRM26-B^c^	NA	NA
23	NA	BREBRM28-B^a,c^	NA	NA
24	NA	BREBRM30-B^a,c,d^	NA	NA
25^&^	NA	BREBRM32-B^a,c^	X	NA
26	NA	BREBRM45-B^a,c^	X	NA
		BREBRM46-B^a,c^	NA	
		BREBRM47-B^a,c^	NA	
27^&^	NA	BREBRM48-B^a,c^	NA	NA
28	NA	BREBRM49-B^a,c,d^	NA	NA
29	NA	BREBRM50-B^c^	NA	NA
30	NA	BREBRM51-B^a,c^	NA	NA
31	NA	BREBRM52-B^a,c^	NA	NA
32	NA	BREBRM53-B^a,c^	NA	NA
33^&^	NA	BREBRM54-B^a,c,d^	NA	NA
34^$,&^	NA	LUNBRM14-B^a,c^	NA	NA
		LUNBRM44-B^a,c^		
35	NA	LUNBRM15-B^a,c^	X	NA
36	NA	LUNBRM17-B^a,c,d^	X	NA
37^$,&^	NA	LUNBRM18-B^a,c^	NA	NA
		LUNBRM36-B^a,c^		
38	NA	LUNBRM27-B^a,c^	X	NA
39^&^	NA	LUNBRM31-B^a,c^	X	NA
40	NA	LUNBRM33-B^a,c^	NA	NA
41	NA	LUNBRM34-B^a,c^	X	NA
42^&^	NA	LUNBRM35-B^a,b,c,d^	X	NA
43^$,&^	NA	LUNBRM37-B^a,c^	NA	NA
44	NA	LUNBRM38-B^a,c^	X	NA
45	NA	LUNBRM39-B^a,c,d^	X	NA
46^&^	NA	LUNBRM40-B^a,c^	X	NA
47	NA	LUNBRM41-B^a,c^	X	NA
48^&^	NA	LUNBRM42-B^a,b,c,d^	X	NA
49	NA	LUNBRM43-B^a,c,d^	X	NA
50^&^	NA	RCCBRM55-B^a,c^	X	NA
51	NA	RCCBRM56-B^a,c^	X	NA
52	NA	RCCBRM57-B^a,c^	NA	NA
53	NA	RCCBRM58-B^a,c^	X	NA
54^&^	NA	RCCBRM59-B^a,c^	X	NA
55	MELBRM1-A^c^	NA	NA	NA
56	MELBRM3-A^c^	NA	NA	NA
57	MELBRM5-A^c^	NA	NA	NA
58	MELBRM6-A^c^	NA	NA	NA
59	MELBRM13-A^c^	NA	NA	NA

*BBM* breast cancer-derived brain metastases, *BrMs* brain metastases, *LBM* lung cancer-derived brain metastases, *MBM melanoma-derived* brain metastases, *PM* primary melanoma, *PT* primary tumour.

List of the specimens derived from primary tumours and BrMs evaluated in the study. Patients indicated with ($) received IO treatment before BrMs diagnosis and/or craniotomy. Patients indicated with (&) received IO treatment after BrMs diagnosis and/or craniotomy.

^a^Subset of tumour specimens subjected to RNA extraction following selection of the tumour area and assessed by RNAseq (*n* = 54 BrMs derived from 50 different patients); BREBRM26-B and BREBRM50-B specimens were not assessed by RNAseq due to the poor QC readouts.

^b^Subset of tumour specimens assessed by Digital Spatial Profile using GeoMx NanoString [*n* = 8 (2 PM, 2 MBM, 2 BBM, 2 LBM), 32 Areas of Illumination in total].

^c^Subset of tumour specimens assessed by IHC (*n* = 56 BrMs derived from 52 different patients and *n* = 7 PM derived from 7 different patients).

^d^Subset of tumour specimens assessed by immunofluorescence (S100B-PMEL17/PanCK, CD3, CD20, NeuN and SYTO13) [*n* = 15 BRMs (5MBM, 5 BBM, 5 LBM)]. Patients indicated with (X) in “Circulating immune cells information” section had available lymphocytes and neutrophil counts assessed in the peripheral blood at the time of brain tumour biopsy collection. Six TIL clones were generated from MBM-TIL-14 fresh tissue specimen and 14 TIL clones were generated from SKCM-TIL-15 fresh tissue specimen.

### Digital spatial profiling (DSP)

High-plex proteomic analyses with spatial resolution were conducted on 56 immune proteins using GeoMx DSP (NanoString Technologies, Seattle, WA, USA) following instructions elsewhere described [19]. FFPE sections were deparaffinized and incubated with a mixture of detection and morphological markers. Statistical comparisons between Areas of Illumination (AOIs) derived from MBM vs non-MBMs specimens were performed applying a linear mixed-effect model (LMM). Differentially expressed proteins were identified by comparing AOIs between the two groups with a significance of ± Log2 Fold Change (FC) ≥ 0.6 and −log10 (*P*) ≥ 1.3. More information regarding FFPE specimens assessed in this study with digital spatial profiling are provided in Table 2.

### Multiplex immunofluorescence (mIF) staining

Serial 5-μm-thick sections from FFPE tumour specimens were stained for the expression of selected markers. More information regarding FFPE specimens assessed in this study with mIF analysis are provided in Table 2.

### Immunohistochemistry (IHC) analysis

Serial 5 μm-thick sections from FFPE tumour specimens were stained for the expression of selected markers. Data were analysed using GraphPad Prism version 7.04 (GraphPad Software Inc., San Diego, CA). More information regarding FFPE specimens assessed in this study with IHC analysis is provided in Table 2.

### Generation of tumour-infiltrating lymphocytes (TILs) and immunophenotyping

Fresh surgically resected tumours derived from primary melanoma and melanoma brain metastases were used for the generation of TIL cultures following a previously described workflow [20]. Immunophenotyping was conducted on fresh TILs using multiparametric spectral flow cytometry. Briefly, TIL cultures were stained using an 11-antibody panel described in Supplementary Materials and Methods. Stained cells were acquired on Cytek^®^ Northern Lights^TM^ flow cytometer, and data were analysed with Spectroflo^®^ software (Cytek Biosciences) and GraphPad Prism version 7.04 (GraphPad Software Inc., San Diego, CA).

### Overall survival analyses of patients

Duration of overall survival (OS) was calculated from the date of detection of brain metastasis to death. Patients without recorded death were censored at the time of the last known clinical follow-up. All survival analyses were carried out in R v.4.2.2.

## Results

### Increased infiltration of NK cells is observed in the TME of primary melanoma vs. melanoma brain metastases

Previous studies have demonstrated the increased immunogenicity of extracranial metastasis (ECM) and primary melanoma (PM) when compared to MBM [14]. Our team also recently performed a multi-omic analysis of a large, real-world melanoma cohort, detecting in MBM a reduced expression of interferon-gamma (IFNγ) and T cell-inflamed signatures when compared to primary cutaneous melanoma (PCM) or extracranial metastases (ECM) [21]. The immunogenicity of MBM vs. PM was further assessed in this study on a small subset of PM and MBM specimens evaluated by protein digital spatial profiling (DSP) or immunohistochemistry (IHC) and by spectral cytometry immunophenotype of multiple clones of tumour-infiltrating lymphocytes (TILs) derived from *n* = 1 PM and *n* = 1 MBM fresh tissues. Results derived from IHC conducted on FFPEs derived from 7 PM vs 13 MBM showed a trend of increased infiltration of CD45+ immune cells in PM vs MBM (*P* = 0.2; Supplementary Fig. 1A). Further results derived from DSP evaluations conducted on 2 matched FFPE specimens (2 PM and 2 MBM derived from the same patients) and immunophenotype of TILs derived from 1 PM vs 1 MBM might also suggest an increased infiltration of dendritic cells (CD80+ cells), NK cells (CD56 + ), and dim-NK cells (CD3−, CD56 + , CD16 + ) in the TME of PM vs MBM (Supplementary Fig. 1B, C). Additional studies are currently ongoing on prospective collected specimens to further validate these preliminary observations and to deeper explore the role played by dim-NKs (CD56 + CD16 + ) and bright-NKs (CD56 + , CD16−) cells in PM and MBM.

### Higher levels of circulating neutrophils and NLR are associated with shorter survival times following craniotomy

**Fig. 1:**
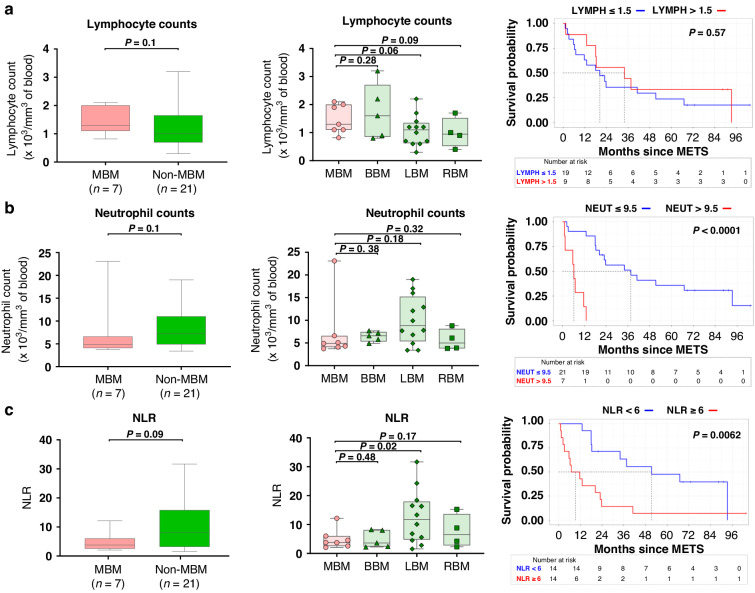
Circulating immune cells repertoire in patients with BrMs. **a** Comparison of lymphocyte counts between MBM and non-MBM assessed when craniotomy. Kaplan–Meier curves were used to illustrate the association between the circulating lymphocyte counts and overall survival (OS) in all patients with BrMs. **b** Comparison of neutrophil counts between MBM and non-MBM assessed when brain tumour biopsy was collected. Kaplan–Meier curves were used to illustrate the association between the circulating neutrophil counts and overall survival (OS) in all patients with BrMs. **c** Comparison of NLR between MBM and non-MBM assessed when brain tumour biopsy was collected. Kaplan–Meier curves were used to illustrate the association between NLR and overall survival (OS) in all patients with BrMs. One-tailed Mann–Whitney tests or one-tailed *t* tests were used to calculate *P* values. OS is here calculated from the time of BrMs diagnosis.

Despite the reduced immunogenicity observed in MBM vs. ECM and PM, we hypothesised MBM to be more immunogenic when compared to BrMs derived from other solid tumours (non-MBM). Acknowledging the multiple evidence suggesting that the immune repertoire in peripheral blood can mirror the immune status of the tumour microenvironment (TME) [22, 23]. we first assessed the lymphocyte counts, the neutrophil counts, and the neutrophil lymphocytes ratio (NLR) detected at the time of craniotomy in the peripheral blood of patients with brain metastasis. The results showed trends of increased circulating lymphocyte counts (*P* = 0.12), as well as decreased neutrophil counts (*P* = 0.12) and decreased NLR (*P* = 0.09) in patients with MBM when compared to patients with non-MBM (Fig. 1a–c**)**. Interestingly, the increased NLR observed in the non-MBM patient’s cohort here analysed seems to be driven by LBM patients (Fig. 1c). Further evaluations also highlighted significant associations between high neutrophil counts, high NLR, and reduced overall survival (OS) of patients following craniotomy, independent of the tumour types under consideration (Fig. 1a–c). These results support previous evidence showing neutrophil counts and NLR to be important peripheral biomarkers associated with the clinical course of patients with brain metastasis [22]. Of note, except for two patients whose corticosteroid treatment information was unavailable, all patients here assessed were receiving corticosteroids prior to blood draw. In addition, none of the patients here evaluated received chemotherapy, radiotherapy or ICB therapies within 30 days preceding the blood collection.

### Whole gene expression profiling reveals distinct neuro-immune architectures in melanoma vs. non-melanoma-derived brain metastasis

**Fig. 2:**
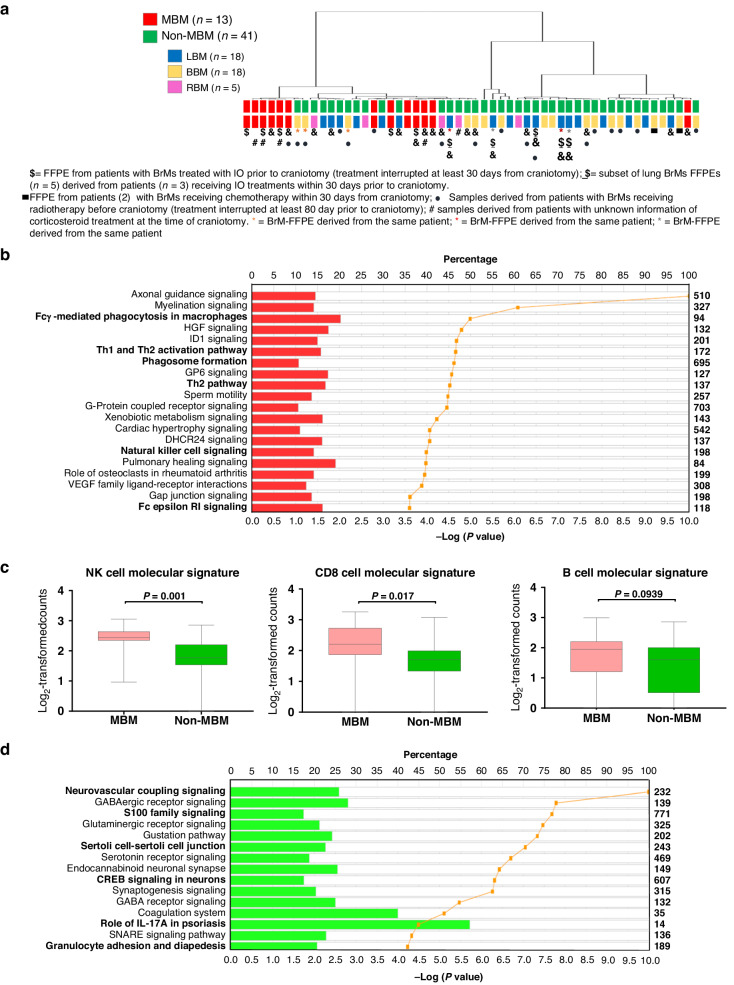
Molecular characterisation of the tumour microenvironment of MBM vs non-MBM. **a** Dendrogram based on the on the whole transcriptome (17912 transcripts) between MBM (red) and other BrMs (green). In (*) are indicated samples derived from the same patient; in ($) are indicated samples derived from patients with BrMs treated with ICB prior to craniotomy; in ($) are indicated subset of samples derived from patients with BrMs treated with ICB within 30 days prior to craniotomy; in (&) are indicated samples derived from patients with BrMs treated with ICB after craniotomy. In (●) are indicated samples derived from patients with BrMs treated with radiotherapy before craniotomy (treatment interrupted at least 80 days prior to craniotomy); in (■) are indicated samples derived from patients with BrMs treated with chemotherapy within 30 days prior to craniotomy. All samples are derived from patients undergoing corticosteroid at the time of craniotomy with the exception of the ones indicated in (#). **b** Top 20 canonical pathways derived from Ingenuity Pathway Analysis (IPA) and associated with the DEG upregulated (adjusted *P* value ≤ 0.05) in MBM vs non-MBM. **c** Representation of the expression level of different molecular signatures (NK, CD8 and B cell) in patients with MBM (red) and non-MBM (green) brain metastasis. One-tailed *t* tests were used to calculate *P* values. **d** Top 15 canonical pathways derived from IPA and associated with the DEG downregulated (adjusted *P* value ≤ 0.05) in MBM vs non-MBM. The “Percentage” value of canonical pathways reported in (**b**, **d**) is calculated as the number of analysis-ready genes in each pathway, divided by the total number of genes in the reference dataset that make up that pathway. The IPA *P* value of canonical pathways reported in (**b**, **d**) are derived from Fisher’s exact test for each pathway and indicates the statistical significance of the overlap of analysis-ready genes that are within the pathway.

To further characterise the TME of patients with MBM and non-MBM, we performed whole gene expression profiling on the tumour regions selected by the pathologist for 54 FFPE specimens derived from 50 unique patients with BrMs originating from different tumours. Unsupervised cluster analysis performed on whole gene expression profiles (17912 transcripts) clearly showed the TME of MBM to cluster differently from the TME of non-MBM **(**Fig. 2a**)**. Additional expression analysis revealed a subset of 4762 genes differentially expressed (adjusted *P* ≤ 0.05) between MBM and non-MBM. Among them, 1841 genes were upregulated, and 2921 genes were downregulated in MBM vs. non-MBM, respectively (Supplementary Table S2). Functional IPA pathway analysis conducted on the subset of 1841 transcripts highlighted that genes upregulated in the TME of MBM vs. non-MBM are involved in Th1 and Th2 activation, Fcg Receptor-mediated phagocytosis in macrophages and monocytes, phagosome formation and Natural Killer cell signalling (Fig. 2b) thus suggesting the TME of MBM to be more immunogenic compared to the TME of non-MBM. Further analysis conducted on the gene expression data indicated the expression of mRNA signatures previously shown by us and others to detect NK cells, CD8 cells and B cells in the TME, and to be associated with clinical response to immune checkpoint blockade (ICB) in solid tumours [24–26], were enriched in the TME of MBM vs. no-MBM (Fig. 2c). Conversely, an increased expression of genes associated with neurodevelopment (*S100A2/4/8/9* and *RBFOX3*, also known as NeuN), cell–cell adhesion (*LAMA3*), Hypoxia (*NOS1*, *HIF1A*), adenosine signalling (*ADORA2B*, *ADORA1*), Th17 pathway (*IL17RB*, *IL23A*), neutrophil enrichment (*CXCL8*, *CCL20*, *CXCR1*), and other immunosuppressive functions previously reported by us and others to be associated with poor response to ICB in solid tumours [27–33] was found highly increased in the TME of non-MBM vs. MBM (Supplementary Table S2 and Fig. 2d).

### Proteomic evaluations conducted by immunohistochemistry and spatial digital pathology highlight increased immunogenicity of MBM vs. non-MBM

**Fig. 3:**
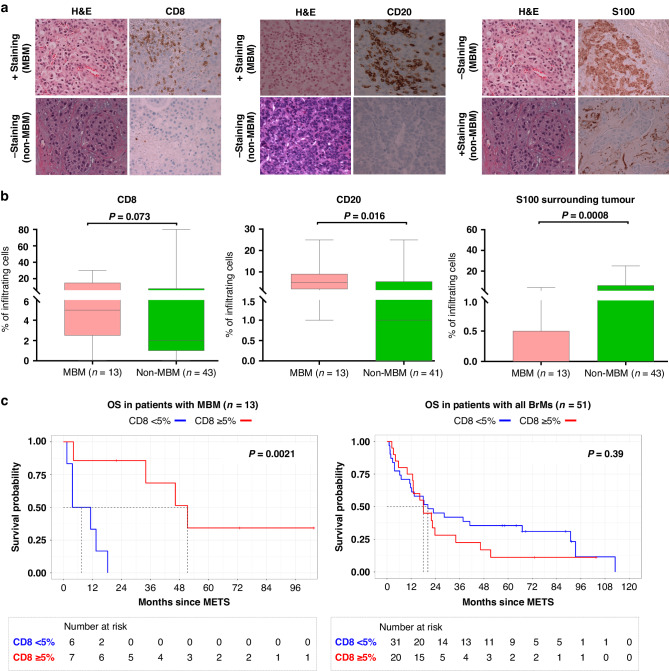
IHC analysis of paraffin-embedded tumour specimens (FFPE) derived from MBM vs non-MBM. Representative data from positive and negative CD8 (**a**), CD20 (**b**) and S100 (**c**) staining’s conducted on specimens derived from MBM and non-MBM patients are shown here with ×40 magnification. Positive and negative CD8^+^ staining cells percentage was 30% and 0% for MBM and non-MBM specimens, respectively (left panel). Positive and negative CD20^+^ staining cells percentage was 25% and 0% for MBM and non-MBM specimens, respectively (medium panel). In MBM representative case, S100^+^ staining cells percentage was 95% (tumoral area) and 0% (stromal area). In non-MBM representative case, S100+ staining cells percentage was 0% (tumoral area) and 25% (stromal area) (right panel). **b** The percentage of CD8^+^ (left panel), CD20^+^ (medium panel) and S100^+^ cells (right panel) evaluated by IHC are shown in MBM vs non-MBM. One-tailed Mann–Whitney tests were used to calculate *P* values. **c** Kaplan–Meier curves assessing the association between CD8^+^ expression in the TME and patients OS in melanoma-derived brain mets (left panel) and in all BrMs (right panel), OS was calculated from BrMs diagnosis to death or to the last patient contact.

Results derived from gene expression analysis were further evaluated at the protein level by conducting immunohistochemistry (IHC) evaluations on 56 FFPE specimens derived from *n* = 52 unique patients with brain metastasis. Specifically, the expression of CD8, CD20 and of the stromal S100 was evaluated by IHC on specimens with available FFPEs, listed in Table 2. Results derived from IHC staining showed a trend of higher density of CD8^+^ cells, higher infiltration of CD20^+^ cells and lower-stromal S100B expression in the TME of MBM compared with non-MBM, respectively (*P* = 0.07, *P* = 0.016, *P* = <0.001, Fig. 3a–c), thus confirming transcriptomic evaluations. Furthermore, Kaplan–Meier (KM) evaluations in patients with MBM patients showed a positive association between a higher density of CD8^+^ cells in the TME and longer OS. Interestingly, no significative association was found between CD8^+^ cells and OS when all the BrMs cases were taken into consideration (Fig. 3c). In addition, no significant association with OS was found when CD20^+^ or stromal S100^+^ cells were taken into consideration (data not shown).

**Fig. 4:**
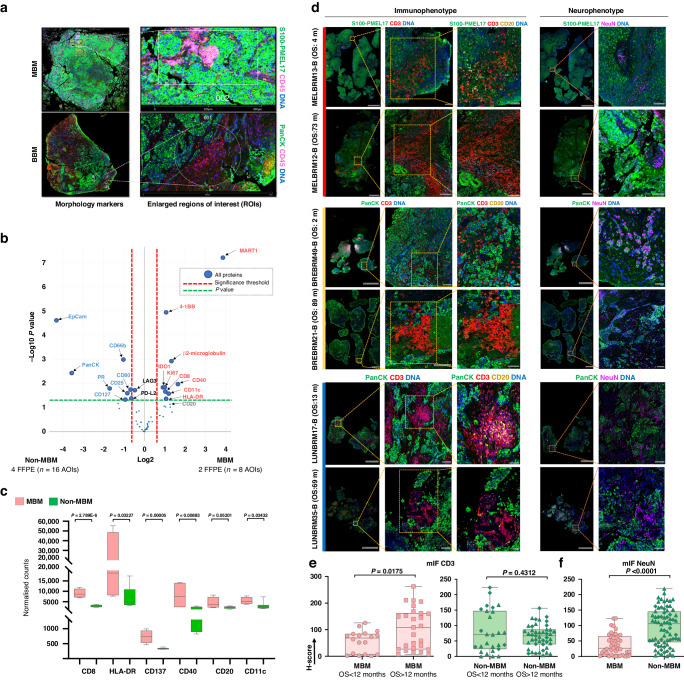
Digital spatial profile based on 56 selected immune-related proteins expressed in MBM vs non-MBM. **a** Representative images of a MBM and a BBM samples. Morphology markers: S100B-PMEL17 /PanCK (green), CD45 (pink) and DNA (blue). **b** Volcano plot representations based on the expression of 56 proteins between 8 bulk Areas of Illumination, (AOIs) derived from MBM vs 16 bulk AOIs derived from non-MBM. Each dot of the graph corresponds to a protein. The fold difference in expression between the different groups is graphed on the *x* axis (logarithm of the base twofold changes). The *P* value for each protein is graphed on the y axis (negative logarithm to the base 10). Dashed lines indicate the threshold of significant protein expression, defined ±Log2 FC ≥ 0.6 and −log10 (*P*) ≥ 1.3 after Lineal Mix Model analysis. Highlighted proteins depicted on the volcano plots are significantly enriched in MBM (right part, red) or non-MBM (left part, blue) in bulk TME (tumour S100B-PMEL17^+^/PanCK^+^ and CD45^+^ compartments). Highlighted in black are other proteins with −log10 (*P*) ≥ 1.3. **c** Representation of CD8, HLA-DR, CD137, CD40, CD20 and CD11c expression between MBM vs non-MBM specimens in the S100B-PMEL^+^/PanCk^+^ tumoral AOIs. *P* values are derived from the Lineal Mix Model analysis between both groups. **d** Representative images of the immunophenotype and neuro-phenotype of two MBM cases (low and high OS post-craniotomy), two BBM cases (low and high OS post-craniotomy) and two LBM (low and high OS post-craniotomy) derived from immunofluorescence staining. OS calculated from BrMs diagnosis to death or to the last patient contact. **e** CD3 H-scores in MBM (left panel), and non-MBM (right panel) stratified by OS (cutoff of 12 months) and evaluated by mIF. In (▲) are indicated samples derived from BBM patients. In (♦) are indicated samples derived from LBM patients. One-tailed Mann–Whitney tests were used to calculate *P* values. **f** NeuN H-scores in MBM vs non-MBM evaluated by mIF. In (▲) are indicated samples derived from BBM patients. In (♦) are indicated samples derived from LBM patients. One-tailed Mann–Whitney tests were used to calculate *P* values.

To deeper explore differences in the tumour immune microenvironment between MBM vs. non-MBM, additional assessments were performed by digital spatial profiling (DSP) in 24 Area of Illumination (AOIs) selected from 6 FFPE specimens assessed by a panel of 56 of selected immune markers. DSP results confirmed the inter-TME heterogeneity between MBM and non-MBM observed by IHC, revealing higher infiltration of CD8^+^ cells, antigen-presenting cells (APCs), (HLA-DR^+^, CD11c^+^, B2M^+^) and B cells (CD20^+^) in MBM vs. non-MBM. Of relevance, agonists of T-cell activity such as CD40 and the surface receptor 4-1BB (CD137, TNFRSF9), known to promote T-cell proliferation, survival, and cytotoxic functions [34], were also strongly increased in MBM vs. non-MBM TME. Conversely, increased infiltration of Tregs (CD25^+^, CD127^+^), neutrophils (CD66b^+^) and epithelial cells (EpCAM^+^, PanCK^+^) was observed in non-MBM compared with MBM (Fig. 4a–c and Supplementary Table S3).

### Immunofluorescence analysis revealed an inverse correlation between CD3+ cells and NeuN+ cells in the TME of patients with MBM

To further map the neural-immune architectures in the TME of BrMs derived from different solid tumours, we next assessed the expression of CD3, CD20, and NeuN (a marker usually associated with neural development) by multiplex immunofluorescence (mIF) analysis. This analysis was conducted in a subset of 15 FFPE specimens derived from patients with MBM (*n* = 5), BBM (*n* = 5) and LBM (*n* = 5) and characterised by highest (*n* = 6), medium (*n* = 3) and lowest (*n* = 6) overall survival following craniotomy.

Results confirmed an increased infiltration of CD3^+^ cells in MBM with higher OS− (Fig. 4e). No significant association between CD3^+^ cells and OS was found looking at the eight cases of brain metastasis derived from BBM and LBM, suggesting the need to further explore the impact and functional status of CD3^+^ (and of CD8^+^ cells) in the TME of these patients (Fig. 4e). Interestingly, regions of interest (ROIs) showing aggregates of CD3^+^ and CD20^+^ cells potentially forming tertiary lymphoid structures (TLS) were observed in brain metastases derived from lung cancer and melanoma patients characterised by medium OS (13 months and 22 months, respectively). However, deeper understanding of the impact of TLS on patients with MBM is currently explored on a larger dataset of patients. Focusing on NeuN+ infiltration, mIF results confirmed an enrichment of NeuN+ cells in the TME of patients with non-MBM vs. MBM (Fig. 4d–f). However, larger sample sizes are needed to accurately evaluate the impact of TLS and NeuN+ cells on the clinical outcome of patients with brain metastasis.

## Discussion

Brain metastases (BrMs) are the most common intracranial malignancy in adults, and a devastating and often fatal complication of melanoma, lung cancer, renal cell carcinoma and breast cancer [1]. Current practice guidelines and management of brain metastases include stereotactic radiosurgery for patients with oligometastatic disease in the brain, and surgery, which is used for highly symptomatic or threatening metastases. Recently, the advent of ICB significantly improved the clinical outcome of patients with brain metastasis, and exceptional results have been obtained, particularly in melanoma [8–10]. Nevertheless, patients with MBM treated with ICB show only a 2.4% OS at 5 years, and the benefit from treatment is observed only in a subset of patients. In addition, limited efficacy of ICB is observed in brain metastasis derived from other solid tumours including lung cancer, breast cancer and renal cell carcinoma, thus suggesting the existence of additional features playing a pivotal role in ICB treatment resistance in brain metastases originating from non-melanoma tumours.

In this study, we evaluated clinical immune-based assessments in the peripheral blood, and we applied complementary molecular and proteomic approaches based on cutting-edge techniques to analyse the TME and the tumour immune microenvironment (TIME) of BrMs derived from different solid tumours.

Particularly, to explore differences versus commonalities in the immunologic characteristics of BrMs arising from different solid tumours, we first assessed differences in peripheral blood absolute lymphocyte, neutrophil counts, and NLR at the time of brain metastasis surgery (craniotomy). Our results showed a slight increase of circulating lymphocytes and a trend of decreased neutrophil counts and NLR in patients with MBM compared to non-MBM patients. Interestingly, increased absolute neutrophil counts and NLR in the peripheral blood were strongly associated with poor OS. This is an important observation that has been reported in many other therapeutic trials as well as retrospective studies, and it emphasizes the utility of monitoring simple parameters like circulating blood counts in the management of patients with BrMs [22]. However, more multicenter studies and larger sample sizes are needed to validate the predictive vs. prognostic impact of NLR on the clinical outcome of patients with brain metastasis.

In the TME, molecular and proteomic evaluations performed on FFPEs further showed that the microenvironment of MBM was highly immunogenic when compared to non-MBM.

Particularly, the MBM tumour microenvironment was characterised by increased infiltration of NK cells, antigen-presenting cells (e.g., dendritic cells, DCs), Th1 cells, and B cells, thus suggesting that a synchronised innate, adaptive, and humoral immune interplay orchestrates higher immunogenicity in brain metastases derived from melanoma. Interestingly, the presence of a functional interplay between NK, DCs, CD8 cells and B cells in the TME has also been shown to be associated with the likelihood of improved responses to ICB in multiple tumour types [26, 35, 36]. In addition, in patients with MBM, increase infiltration of CD8^+^ cells detected in the TME at the time of craniotomy was associated with better overall survival following surgical resection. Of note, 18 out of 53 patients with brain metastasis here evaluated and derived from different tumour types (6 MBM, 7 LBM, 3 BBM, 2 RBM), received ICB treatments following craniotomy. Unfortunately, no clear associations with ICB response can be evaluated in this retrospective study due to the limited number of patients available in each subgroup and the variety of additional treatments received after craniotomy in addition to ICB. Despite this limitation, the results here obtained could altogether suggest that the increased response to ICB observed in MBM might potentially be due to the increased immunogenicity of MBM vs. other BrMs and that other studies are critical to the development of more appropriate treatment interventions for non- melanoma BrMs.

Interestingly, the tumour microenvironment of MBM was also observed to be highly enriched with T-cell co-stimulatory signals such as CD40 and CD137, suggesting that strategies that include agonistic co-stimulation of these receptors might increase ICB response and overall survival in patients with MBM.

Focusing on non-melanoma-derived brain metastases, the expression of genes and proteins associated with neurodevelopment, cell–cell adhesion, immune suppression, and other functions previously shown to be associated with poor response to ICB in solid tumours [27–33] was found highly increased in the TME of non-MBM vs. MBM brain metastases.

These results provide the basis to further explore the impact of the TME neuro-immune architectures on the regulation of treatment resistance and impairment of anti-cancer immunity in patients with brain metastases. In addition, although the identification of distinct features associated with ICB response of brain metastases is not the focus of this retrospective study, these data might suggest that pathways associated with neurodevelopment and immunosuppressive functions that were found enriched in non-MBM vs. MBM could potentially lead to a limited response to ICB in patients with non- MBM brain metastases.

## Conclusions

While these exploratory findings require further confirmation in larger independent cohorts, our results provide the basis for the first comprehensive transcriptomic and proteomic immune atlas characterisation of BrMs arising from different solid tumours. This study also highly supports the development of follow-up studies to mechanistically explore our findings and guide new translational therapeutic research for patients with solid tumours metastatic to the brain.

## Supplementary information

Supplementary figure 1

Supplementary figure 2

Supplementary table 1

Supplementary table S2

Supplementary table S3

Supplementary Materials and Methods

## Data Availability

Gene expression data discussed in this publication have been deposited in NCBI’s Gene Expression Omnibus [37] and are accessible through GEO Series accession number GSE245467.

## References

[1] Seoane J, De Mattos-Arruda L. Brain metastasis: new opportunities to tackle therapeutic resistance. Mol Oncol. 2014;8:1120–31. 24953014 10.1016/j.molonc.2014.05.009 PMC5528619

[2] Amsbaugh MJ, Kim CS. Brain Metastasis. In: StatPearls [Internet]. Treasure Island (FL): (StatPearls Publishing; 2024). 29262201

[3] Campbell BK, Gao Z, Corcoran NM, Stylli SS, Hovens CM. Molecular mechanisms driving the formation of brain metastases. Cancers. 2022;14:4963. 36230886 10.3390/cancers14194963 PMC9563727

[4] Sloan AE, Nock CJ, Einstein DB. Diagnosis and treatment of melanoma brain metastasis: a literature review. Cancer Control. 2009;16:248–55. 19556965 10.1177/107327480901600307

[5] Stelzer KJ. Epidemiology and prognosis of brain metastases. Surg Neurol Int. 2013;4:S192. 23717790 10.4103/2152-7806.111296 PMC3656565

[6] Klemm F, Maas RR, Bowman RL, Kornete M, Soukup K, Nassiri S, et al. Interrogation of the microenvironmental landscape in brain tumors reveals disease-specific alterations of immune cells. Cell. 2020;181:1643–60.e17. 32470396 10.1016/j.cell.2020.05.007 PMC8558904

[7] Srinivasan ES, Deshpande K, Neman J, Winkler F, Khasraw M. The microenvironment of brain metastases from solid tumors. Neuro-oncol Adv. 2021;3:v121–v32. 10.1093/noajnl/vdab121 PMC8633769 34859239

[8] Tawbi HA, Forsyth PA, Algazi A, Hamid O, Hodi FS, Moschos SJ, et al. Combined nivolumab and ipilimumab in melanoma metastatic to the brain. New Engl J Med. 2018;379:722–30. 30134131 10.1056/NEJMoa1805453 PMC8011001

[9] Tawbi HA, Forsyth PA, Hodi FS, Algazi AP, Hamid O, Lao CD, et al. Long-term outcomes of patients with active melanoma brain metastases treated with combination nivolumab plus ipilimumab (CheckMate 204): final results of an open-label, multicentre, phase 2 study. Lancet Oncol. 2021;22:1692–704. 34774225 10.1016/S1470-2045(21)00545-3 PMC9328029

[10] Goldberg SB, Schalper KA, Gettinger SN, Mahajan A, Herbst RS, Chiang AC, et al. Pembrolizumab for management of patients with NSCLC and brain metastases: long-term results and biomarker analysis from a non-randomised, open-label, phase 2 trial. Lancet Oncol. 2020;21:655–63. 32251621 10.1016/S1470-2045(20)30111-X PMC7380514

[11] Schmid P, Adams S, Rugo HS, Schneeweiss A, Barrios CH, Iwata H, et al. Atezolizumab and nab-paclitaxel in advanced triple-negative breast cancer. New Engl J Med. 2018;379:2108–21. 30345906 10.1056/NEJMoa1809615

[12] Frisone D, Friedlaender A, Addeo A, Tsantoulis P. The landscape of immunotherapy resistance in NSCLC. Front Oncol. 2022;12:817548. 10.3389/fonc.2022.817548 PMC9066487 35515125

[13] Gide TN, Wilmott JS, Scolyer RA, Long GV. Primary and acquired resistance to immune checkpoint inhibitors in metastatic melanomaresistance to immunotherapy in melanoma. Clin Cancer Res. 2018;24:1260–70. 29127120 10.1158/1078-0432.CCR-17-2267

[14] Fischer GM, Jalali A, Kircher DA, Lee W-C, McQuade JL, Haydu LE, et al. Molecular profiling reveals unique immune and metabolic features of melanoma brain metastases. Cancer Discov. 2019;9:628–45. 30787016 10.1158/2159-8290.CD-18-1489 PMC6497554

[15] Schoenfeld DA, Moutafi M, Martinez S, Djureinovic D, Merkin RD, Adeniran A, et al. Immune dysfunction revealed by digital spatial profiling of immuno-oncology markers in progressive stages of renal cell carcinoma and in brain metastases. J Immunother Cancer. 2023;11:e007240. 37586773 10.1136/jitc-2023-007240 PMC10432651

[16] Lu BY, Gupta R, Aguirre-Ducler A, Gianino N, Wyatt H, Ribeiro M, et al. Spatially resolved analysis of the T cell immune contexture in lung cancer-associated brain metastases. J Immunother Cancer. 2021;9:e002684. 34670827 10.1136/jitc-2021-002684 PMC8529973

[17] Ascierto ML, McMiller TL, Berger AE, Danilova L, Anders RA, Netto GJ, et al. The intratumoral balance between metabolic and immunologic gene expression is associated with anti-PD-1 response in patients with renal cell carcinoma. Cancer Immunol Res. 2016;4:726–33. 27491898 10.1158/2326-6066.CIR-16-0072 PMC5584610

[18] Robinson MD, McCarthy DJ, Smyth GK. edgeR: a Bioconductor package for differential expression analysis of digital gene expression data. Bioinformatics. 2010;26:139–40. 19910308 10.1093/bioinformatics/btp616 PMC2796818

[19] Merritt CR, Ong GT, Church SE, Barker K, Danaher P, Geiss G, et al. Multiplex digital spatial profiling of proteins and RNA in fixed tissue. Nat Biotechnol. 2020;38:586–99. 32393914 10.1038/s41587-020-0472-9

[20] Jin J, Sabatino M, Somerville R, Wilson JR, Dudley ME, Stroncek DF, et al. Simplified method of the growth of human tumor infiltrating lymphocytes (TIL) in gas-permeable flasks to numbers needed for patient treatment. J Immunother. 2012;35:283. 22421946 10.1097/CJI.0b013e31824e801f PMC3315105

[21] In GK, Ribeiro JR, Yin J, Xiu J, Bustos MA, Ito F, et al. Multi-omic profiling reveals discrepant immunogenic properties and a unique tumor microenvironment among melanoma brain metastases. NPJ Precis Oncol. 2023;7:120. 37964004 10.1038/s41698-023-00471-z PMC10646102

[22] Koh YW, Choi J-H, Ahn MS, Choi YW, Lee HW. Baseline neutrophil–lymphocyte ratio is associated with baseline and subsequent presence of brain metastases in advanced non-small-cell lung cancer. Sci Rep. 2016;6:38585. 27924837 10.1038/srep38585 PMC5141478

[23] Choi Y, Kim JW, Nam KH, Han S-H, Kim J-W, Ahn S-H, et al. Systemic inflammation is associated with the density of immune cells in the tumor microenvironment of gastric cancer. Gastric Cancer. 2017;20:602–11. 27665104 10.1007/s10120-016-0642-0

[24] Mendoza-Valderrey A, Alvarez M, De Maria A, Margolin K, Melero I, Ascierto ML. Next generation immuno-oncology strategies: unleashing NK cells activity. Cells. 2022;11:3147. 36231109 10.3390/cells11193147 PMC9562848

[25] Böttcher JP, Bonavita E, Chakravarty P, Blees H, Cabeza-Cabrerizo M, Sammicheli S, et al. NK cells stimulate recruitment of cDC1 into the tumor microenvironment promoting cancer immune control. Cell. 2018;172:1022–37.e14. 29429633 10.1016/j.cell.2018.01.004 PMC5847168

[26] Helmink BA, Reddy SM, Gao J, Zhang S, Basar R, Thakur R, et al. B cells and tertiary lymphoid structures promote immunotherapy response. Nature. 2020;577:549–55. 31942075 10.1038/s41586-019-1922-8 PMC8762581

[27] Ascierto ML, Makohon-Moore A, Lipson EJ, Taube JM, McMiller TL, Berger AE, et al. Transcriptional mechanisms of resistance to anti-PD-1 therapy transcriptional mechanisms of resistance to anti-PD-1. Clin Cancer Res. 2017;23:3168–80. 28193624 10.1158/1078-0432.CCR-17-0270 PMC5474192

[28] Liu Q, Tomei S, Ascierto ML, De Giorgi V, Bedognetti D, Dai C, et al. Melanoma NOS1 expression promotes dysfunctional IFN signaling. J Clin Investig. 2014;124:2147–59. 24691438 10.1172/JCI69611 PMC4001531

[29] Chen S, Fan J, Zhang M, Qin L, Dominguez D, Long A, et al. CD73 expression on effector T cells sustained by TGF-β facilitates tumor resistance to anti-4-1BB/CD137 therapy. Nat Commun. 2019;10:150. 30635578 10.1038/s41467-018-08123-8 PMC6329764

[30] Mastelic-Gavillet B, Navarro Rodrigo B, Décombaz L, Wang H, Ercolano G, Ahmed R, et al. Adenosine mediates functional and metabolic suppression of peripheral and tumor-infiltrating CD8+ T cells. J Immunother Cancer. 2019;7:1–16. 31601268 10.1186/s40425-019-0719-5 PMC6788118

[31] Fong L, Hotson A, Powderly JD, Sznol M, Heist RS, Choueiri TK, et al. Adenosine 2A receptor blockade as an immunotherapy for treatment-refractory renal cell cancer. Cancer Discov. 2020;10:40–53. 31732494 10.1158/2159-8290.CD-19-0980 PMC6954326

[32] Duffield AS, Ascierto ML, Anders RA, Taube JM, Meeker AK, Chen S, et al. Th17 immune microenvironment in Epstein-Barr virus-negative Hodgkin lymphoma: implications for immunotherapy. Blood Adv. 2017;1:1324–34. 29296775 10.1182/bloodadvances.2017007260 PMC5727974

[33] Pore N, Wu S, Standifer N, Jure-Kunkel M, de Los Reyes M, Shrestha Y, et al. Resistance to durvalumab and durvalumab plus tremelimumab is associated with functional STK11 mutations in non-small-cell lung cancer patients and is reversed by STAT3 knockdown. Cancer Discov. 2021;11:2828–45. 34230008 10.1158/2159-8290.CD-20-1543

[34] Etxeberria I, Glez-Vaz J, Teijeira Á, Melero I. New emerging targets in cancer immunotherapy: CD137/4-1BB costimulatory axis. ESMO Open. 2019;4:e000733. 10.1136/esmoopen-2020-000733 PMC7333812 32611557

[35] Cabrita R, Lauss M, Sanna A, Donia M, Skaarup Larsen M, Mitra S, et al. Tertiary lymphoid structures improve immunotherapy and survival in melanoma. Nature. 2020;577:561–5. 31942071 10.1038/s41586-019-1914-8

[36] Kießler M, Plesca I, Sommer U, Wehner R, Wilczkowski F, Müller L, et al. Tumor-infiltrating plasmacytoid dendritic cells are associated with survival in human colon cancer. J Immunother Cancer. 2021;9:e001813. 10.1136/jitc-2020-001813 PMC7993360 33762320

[37] Edgar R, Domrachev M, Lash AE. Gene Expression Omnibus: NCBI gene expression and hybridization array data repository. Nucleic Acids Res. 2002;30:207–10. 11752295 10.1093/nar/30.1.207 PMC99122

