# Supplementary material for: Immunogenomics and spatial proteomic mapping highlight distinct neuro-immune architectures in melanoma vs. non-melanoma-derived brain metastasis
Source: BJC Rep. 2024 May 2;2:38. doi: 10.1038/s44276-024-00060-y (PMC11524107; doi:10.1038/s44276-024-00060-y)
Supplement: Supplementary file 1 — Supplementary figure 1 [file 44276_2024_60_MOESM1_ESM.pptx]

## Slide 1
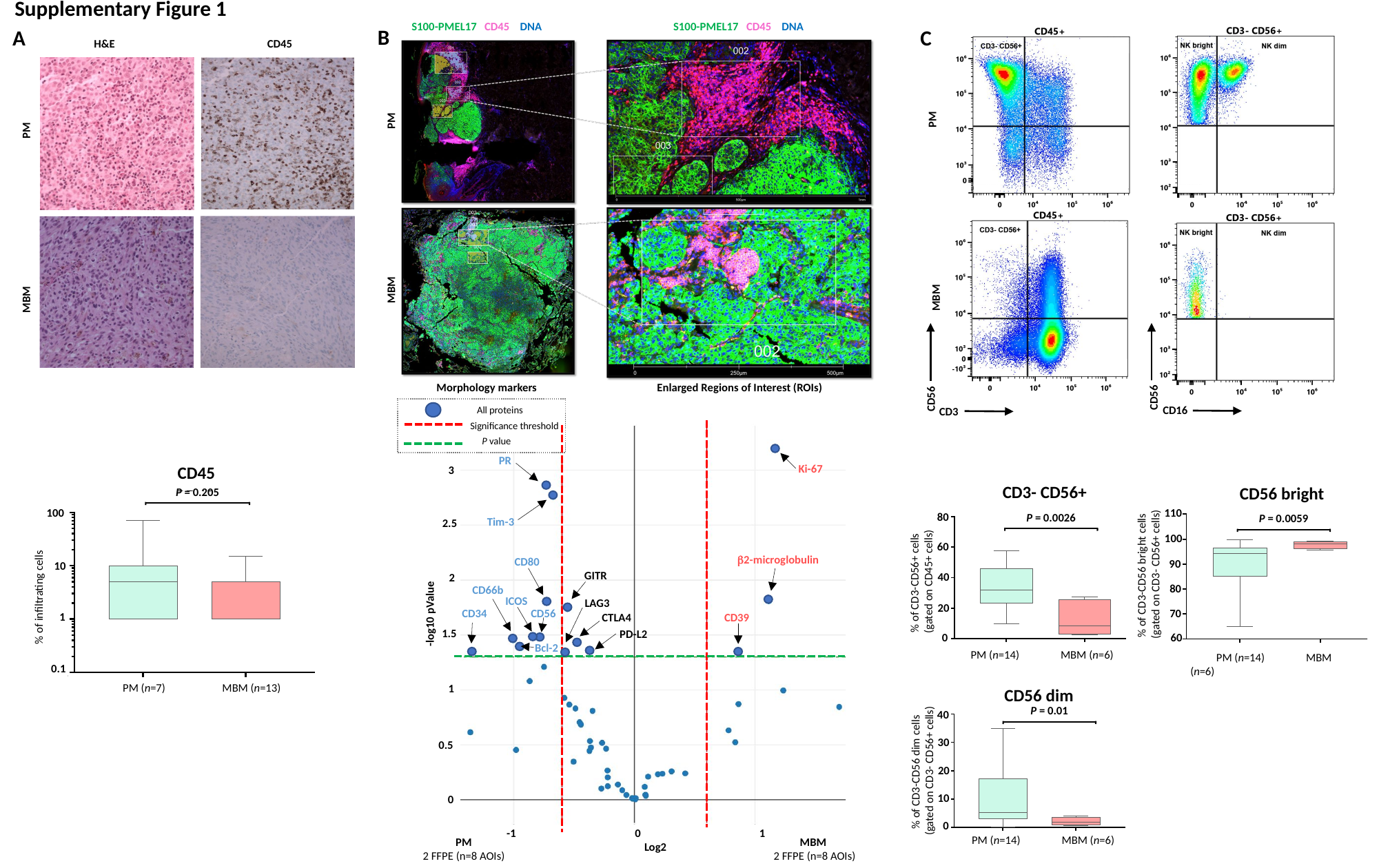

Supplementary Figure 1
S100-PMEL17 CD45 DNA
S100-PMEL17 CD45 DNA
B
C
A
 H&E CD45
PM
PM
PM
MBM
MBM
MBM
Morphology markers
Enlarged Regions of Interest (ROIs)
CD56
CD56
CD16
All proteins
Significance threshold
P value
CD3
PR
Ki-67
CD45
3
CD3- CD56+
CD56 bright
P = 0.205
100
110
80
P = 0.0026
P = 0.0059
Tim-3
2.5
100
60
b2-microglobulin
CD80
90
10
% of CD3-CD56 bright cells
(gated on CD3- CD56+ cells)
% of CD3-CD56+ cells
(gated on CD45+ cells)
GITR
40
2
80
CD66b
% of infiltrating cells
ICOS
LAG3
20
70
CD56
CD34
-log10 pValue
1
CTLA4
CD39
1.5
PD-L2
0
60
Bcl-2
 PM (n=14) MBM (n=6)
 PM (n=14) MBM (n=6)
0.1
 PM (n=7) MBM (n=13)
1
CD56 dim
P = 0.01
40
30
0.5
% of CD3-CD56 dim cells
(gated on CD3- CD56+ cells)
20
10
0
0
 -1 0 1
Log2
 PM (n=14) MBM (n=6)
MBM
2 FFPE (n=8 AOIs)
PM
2 FFPE (n=8 AOIs)
