# Supplementary material for: Immunogenomics and spatial proteomic mapping highlight distinct neuro-immune architectures in melanoma vs. non-melanoma-derived brain metastasis
Source: BJC Rep. 2024 May 2;2:38. doi: 10.1038/s44276-024-00060-y (PMC11524107; doi:10.1038/s44276-024-00060-y)
Supplement: Supplementary file 2 — Supplementary figure 2 [file 44276_2024_60_MOESM2_ESM.pptx]

## Slide 1
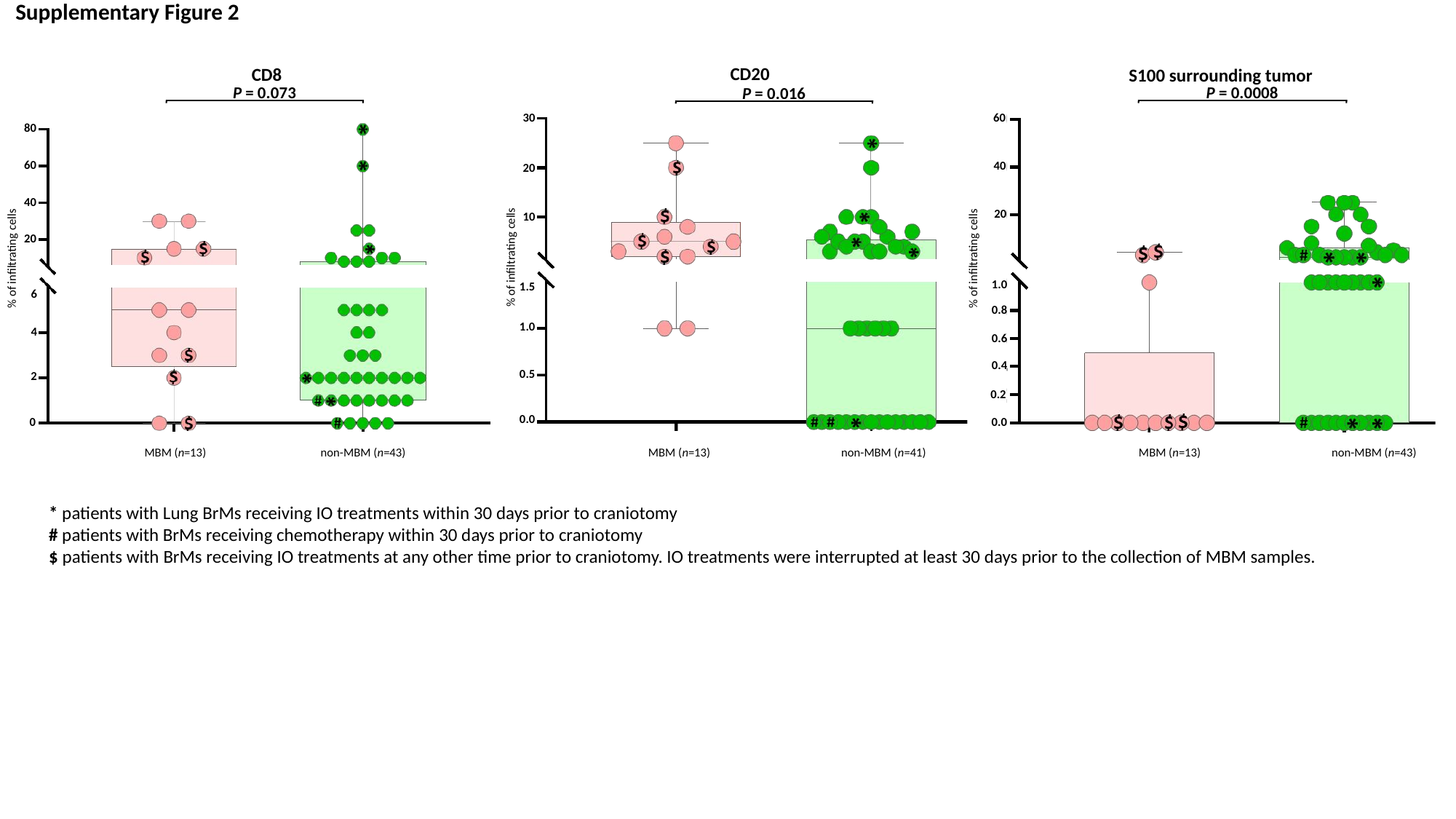

Supplementary Figure 2
CD20
CD8
S100 surrounding tumor
P = 0.073
P = 0.0008
P = 0.016
30
60
80
60
40
20
40
20
10
20
% of infiltrating cells
% of infiltrating cells
% of infiltrating cells
1.0
1.5
6
0.8
1.0
4
0.6
0.4
0.5
2
0.2
0.0
0
0.0
 MBM (n=13) non-MBM (n=43) MBM (n=13) non-MBM (n=41) MBM (n=13) non-MBM (n=43)
* patients with Lung BrMs receiving IO treatments within 30 days prior to craniotomy
# patients with BrMs receiving chemotherapy within 30 days prior to craniotomy
$ patients with BrMs receiving IO treatments at any other time prior to craniotomy. IO treatments were interrupted at least 30 days prior to the collection of MBM samples.
