# Supplementary material for: Immunogenomics and spatial proteomic mapping highlight distinct neuro-immune architectures in melanoma vs. non-melanoma-derived brain metastasis
Source: BJC Rep. 2024 May 2;2:38. doi: 10.1038/s44276-024-00060-y (PMC11524107; doi:10.1038/s44276-024-00060-y)
Supplement: Supplementary file 3 — Supplementary table 1 [file 44276_2024_60_MOESM3_ESM.doc]

| SUPPLEMENTARY Table 1. Patient Characteristics | | |
| --- | --- | --- |
| Total Number SJCI Patients evaluated |  | 59 (53 BRMS patients, 6 BRMS free patients) |
| MEAN AGE AT DIAGNOSIS OF brms (Years) |  | 63.4 |
| gENDER PATIENTS (% out of all patients) |  |  |
| FEMALE |  | 34 (57.6%) |
| MALE |  | 25 (42.4%) |
| PATIENTS with MATCHED BRM-primary tumors (PT) |  | 2 (derived from melanoma patients) |
| PATIENTS with Brain metastasis (BrMs) |  | 53* |
| melanoma- DERIVED BRm (MBM) |  | 14 (26.4%) |
| Lung CA- DERIVED BRm (LBM) |  | 16 (30.2%) |
| Breast CA- DERIVED brm (BBM) |  | 18 (34%) |
| RCC-DERIVED Brm (RBM) |  | 5 (9.4%) |
| Treatment received before brms DIAGNOSIS  (% out of ALL brms PATIENTS) |  |  |
| surgery |  | 35 (66%) |
| chemotherapy |  | 22 (41.5%) |
| radiotherapy |  | 16 (30.19%) |
| immunotherapy |  | 8 (15.1 %) (5 MBM, 3 LBM) |
| OTHERS (I. E. her2 TARGETED THERAPY) |  | 19 (35.8%) (1 LBM, 16 BBM, 2 RBM) |
| UNTREATED |  | 7 (13.2%) |
| Unknown |  | 3 (5.7%) |
| treatment received after brms diagnosis  (% out of ALL brms PATIENTS) |  |  |
| surgery |  | 51 (96.2 %) |
| chemotherapy |  | 17 (32.1%) |
| radiotherapy |  | 46 (86.8%) |
| immunotherapy |  | 18 (34%) (6 MBM, 7 LBM, 3 BBM, 2 RBM) |
| OTHERS (I.E. her2 TARGETED THERAPY) |  | 22 (41.5 %) (3 MBM, 2 LBM, 14 BBM, 3 RBM) |
| Interval between PT and brms diagnosis |  |  |
| <1 year |  | 13 (24.5%) |
| ≥1 year≤ 2 years |  | 13 (24.5%) |
| >2 years |  | 26 (49.1%) |
| UNKNOWN |  | 1 (1.9%) |
| mean overall survival from diagnosis of brms (months) |  | 31.52 |
| mbm |  | 27.61 |
| LBM |  | 20.05 |
| BBM |  | 47.42 |
| RBM |  | 19.58 |
| Patients with Oncogenic mutations |  |  |
| *MUTATIONS IN MAPK PATHWAY (BRAF, RAS)* |  |  |
| POSITIVE |  | 6 (5 MBM, 1 LBM) |
| NEGATIVE |  | 11 (4 MBM, 7 LBM) |
| CASES NOT TESTED |  | 13 (5 MBM, 8 LBM) |
| *mutations in egfr* |  |  |
| POSITIVE |  | 1 LBM |
| NEGATIVE |  | 14 LBM |
| CASES NOT TESTED |  | (1 LBM, 14 MBM) |
| BBM phenotype |  |  |
| HEr2+ |  | 11 BBM |
| ER+ |  | 10 BBM |

**Note:** *Total number of brain metastasis specimens evaluated= 57 from 53 different patients; three BBM specimens from the same pt and four LBM specimens derived from two different pts (2 specimens/each), respectively, were here evaluated. Seven FFPE tissue specimens derived from primary melanoma (PM) were also analyzed in this study. Two PM FFPE tissue specimens had matching brain metastases. One MBM and one PM fresh tissue specimens used for TIL generation and spectral flow cytometry analysis. Information regarding specimens used for the different assessments conducted in this study are provided in **Table 2**.
