# Supplementary material for: Immunogenomics and spatial proteomic mapping highlight distinct neuro-immune architectures in melanoma vs. non-melanoma-derived brain metastasis
Source: BJC Rep. 2024 May 2;2:38. doi: 10.1038/s44276-024-00060-y (PMC11524107; doi:10.1038/s44276-024-00060-y)
Supplement: Supplementary file 6 — Supplementary Materials and Methods [file 44276_2024_60_MOESM6_ESM.doc]

**Supplementary Materials and Methods**

**Patient cohort, specimens and treatments**

Samples derived from 59 unique patients (53 with BrMs and 6 with Primary Melanoma) treated at the Saint John’s Health Center (Santa Monica, CA) between 2004 and 2023 were evaluated in this study using different approaches.

In details, serial 5-μm-thick sections from formalin-fixed, paraffin-embedded (FFPE) specimens from 13 melanoma- derived brain metastasis (MBM), 20 breast cancer- derived brain metastasis (BBM), 18 lung cancer- derived brain metastasis (LBM) and 5 renal cancer- derived brain metastasis (RBM) were obtained from BrMs patients undergoing craniotomy. Except for 3 cases, all brain metastasis FFPEs specimens were derived from unique patients leading to a total of FFPE specimens assessed = 56 from 52 unique patients with BrMs. In addition to FFPEs derived from BrMs, n=7 FFPEs tissue specimens derived from primary melanoma (PM), were also analyzed in this study. Two of the PM cases included had matching FFPEs derived from brain metastases collected at time of craniotomy Two fresh tissues derived from one MBM and one PM independent cases were used for TIL generation and spectral flow cytometry analysis. Blood derived from twenty-eight patients (7 from MBM, 5 from BBM, 12 from LBM, 4 from RBM), were collected at the time of craniotomy and were here- evaluated for lymphocyte and neutrophil counts. Except for 5 cases, all patients with brain metastasis included in this study were treated with corticosteroid at the time of craniotomy. Additionally, a subset of patients with brain metastasis received other form of treatments (chemotherapy, ICB, or radiotherapy) before craniotomy. However, except for 2 patients with BBM and 3 patients with LBM (patients IDs #30, #31, #34, #37, #43 receiving ICB or chemotherapy), all treatments were interrupted at least 30 days prior to craniotomy. More information regarding patients’ treatments is included in Table 1, Supplementary Table 1 and Figure 2A. Additional information regarding specimens used for the different analysis in this study are provided in **Table 2**.

**mRNA gene expression profiling and analysis**

In details, n=56 metastatic FFPE specimens from BrMs were subjected to RNA extraction following selection of the tumor area, identified by a pathologist, and manually scraped as previously described(1). The quality and integrity of extracted RNA was assessed using the Eukaryote Total RNA Pico Kit on the Agilent 2100 Bioanalyzer (Agilent Technologies, Santa Clara, CA). Two samples (BREBRM26B and BREBRM50-B) were discarded after RNAseq Quality Control (QC) assessments leading to a final number of 54 specimens derived from 50 unique patients analyzed by RNAseq.Ribosomal RNA (rRNA) was depleted and RNA-seq libraries were prepared from the 54 specimens using KAPA Stranded RNA-Seq Kit with RiboErase Kit (Roche). Single-indexed libraries were pooled and sequenced on the Illumina NovaSeq 6000 platform for PE 2x100 run. For samples BREBRM21-B, BREBRM32-B and LUNBRM15-B, six available FASTQ files were combined together before proceeding with data analysis (BREBRM21-B_S27_L003_R1_001.fastq.gz, BREBRM21-B_S27_L003_R2_001.fastq.gz, BREBRM21-B_S27_L004_R1_001.fastq.gz, BREBRM21-B_S27_L004_R2_001.fastq.gz, BREBRM21-B_S49_L003_R1_001.fastq.gz, and BREBRM21-B_S49_L003_R2_001.fastq.gz files for sample BREBRM21-B; BREBRM32-B_S25_L003_R1_001.fastq.gz, BREBRM32-B_S25_L003_R2_001.fastq.gz, BREBRM32-B_S25_L004_R1_001.fastq.gz, BREBRM32-B_S25_L004_R2_001.fastq.gz, BREBRM32-B_S43_L003_R1_001.fastq.gz and BREBRM32-B_S43_L003_R2_001.fastq.gz files for sample BREBRM32-B; LUNBRM15-B_S22_L003_R1_001.fastq.gz, LUNBRM15-B_S22_L003_R2_001.fastq.gz, LUNBRM15-B_S22_L004_R1_001.fastq.gz, LUNBRM15-B_S22_L004_R2_001.fastq.gz, LUNBRM15-B_S35_L003_R1_001.fastq.gz and LUNBRM15-B_S35_L003_R2_001.fastq.gz files for sample LUNBRM15-B). Data quality check was done on Illumina SAV. Demultiplexing was performed with Illumina Bcl2fastq v2.19.1.403 software. Initial analysis included alignment with TopHat2(2), and gene expression counts were obtained using cufflinks. FastQC was used for quality control. Dendrograms were generated using MetaboAnalyst (3). IPA software was used to interpret the biological changes, altered canonical pathways and upstream transcriptional regulators.

**Molecular signatures analysis**

Expression counts derived from RNAseq data were obtained for the genes included in the following molecular signatures: B cells (4) (*CD19*, *CXCL13*), CD8+ T cells (*CD8A*, *CD8B*), and NK cells (5, 6) (*KLRD1*, *GNLY*, *KLRC3*, *NCR*1 *KLRF1*) and were assessed in all MBM and all non-MBM. The log2 of median values over all the genes in each signature were displayed separately for MBM and non-MBM patients via a dot plot using GraphPad Prism version 7.04 (GraphPad Software Inc., San Diego, CA). T-tests were used to calculate p values.

**Digital Spatial Profiling (DSP)**

A total number of 6 FFPEs specimens derived from BrMs (2 from MBM, 2 from LBM and 2 from BBM) and 2 FFPEs derived from PM (primary melanoma) were subjected to DSP analysis. None of the primary melanoma tissues evaluated by DSP derived from patients previously treated. Except for one MBM patient with no available information, all other BrMs FFPEs specimens assessed by DSP derived from patients receiving corticosteroids at time of craniotomy. Additionally, three out of six BrMs FFPEs specimens evaluated by DSP derived from patients who received chemotherapy, radiotherapy or ICB treatment prior to craniotomy. Specifically, one FFPE derived from a patient with MBM receiving ICB prior to craniotomy; one FFPE derived from a patient with MBM receiving radiotherapy before craniotomy; one FFPE assessed by DSP derived from a patient with LBM receiving radiotherapy before craniotomy. However, for all three patients here assessed, treatments were interrupted at least 80 days before samples collection.

In conducting DSP evaluations, n=24 Areas of Illumination (AOIs) derived from 6 FFPE specimens from BrMs and n=8 AOIs derived from 2 FFPE specimens from PM were analyzed. Of note, the selection of BrMs cases derived from different tumor types for DSP profiling was made based on OS, independently from treatment received prior or after craniotomy. In details, FFPEs specimens with highest OS and lowest OS derived from each tumor type was assessed. Additionally, also two FFPEs derived from PM with matched MBM FFPEs available were assessed by DSP. Morphological markers -used to visualize tissue compartments and regions of interest (ROIs)- included Syto13 for nuclei, pan-cytokeratin (PanCk) or S100B-PMEL17 for tumor and CD45 for leukocytes. The detection of 59 antibodies including one core panel and five modules of the GeoMx assay including 56 immune (GeoMx immune cell profiling panel, GeoMx IO drug target module, GeoMx immune activation status module, GeoMx immune cell typing module, GeoMx pan-tumor module and GeoMx myeloid module) markers was assessed by DSP. Detailed information about 56 immune markers assessed by DSP is included in the **Supplementary Table S3**.

For each slide, three ROIs, located within the tumor regions annotated in the H&E slides were selected: one ROI located in an exclusively PanCk^+^/ S100B-PMEL17^+^ tumoral area; one ROI in a exclusively CD45^+^ immune infiltrated area and the third ROI in a combined tumoral & immune infiltrated area (PanCk^+^/ S100B-PMEL17^+^ and CD45^+^ region). Each ROI was UV-illuminated twice, once for the PanCk/ S100B-PMEL17 segment and once for the CD45 segment. 4 AOIs were collected per slide: 2 PanCK^+^/ S100B-PMEL17^+^ tumor AOIs and 2 CD45^+^ immune infiltrated stromal AOIs. Photocleaved oligonucleotides from each spatially resolved AOI were PCR amplified. PCR products were pooled and purified twice with AMPure XP beads (Beckman Coulter, Brea, CA). The quality of the final pool library was checked with Agilent TapeStation 4200 system (Agilent Technologies, Santa Clara, CA), and Qubit Flourometer 4.0 (Invitrogen, Waltham, MA), then denatured, normalized, and sequenced with the NextSeq 550 platform with paired-end 27 cycles to generate FASTQ files. Once FASTQ files were generated, the files were converted to DCC files using the BaseSpace GeoMx NGS Pipeline v2.0.21 (Illumina, San Diego, CA) to be compatible with GeoMx DSP Control Center v2.4.2.2. Digital counts between AOIs were normalized with three IgG negative control isotypes (Ms IgG1, Ms IgG2a, Rb IgG). Statistical comparisons between AOIs/group were performed S applying a linear mixed-effect model (LMM) to account for multiple sampling of AOI segments per tissue.

**Multiplex Immunofluorescence (mIF) staining and evaluation of immunofluorescence intensity**

The expression markers CD20-AF594 (NovusBio, clone IGEL/773), CD3-AF532 (NovusBio, clone C3e/1308), CD8a-AF594 (Biolegend, clone C8/144B) and NeuN-AF647 (Abcam, clone EPR12763) were assessed to visualize B cells, T cells and neurons respectively in 15 brain-metastatic FFPEs. Two FFPEs derived from LBB patients were further discarded from mIF quantitative analysis due the significant necrotic content present in the FFPE thus leading to a final number of 13 FFPEs specimens (5 from MBM, 3 from LBM and 5 from BBM) assessed by mIF. and Briefly, a 20X scan image of each brain metastatic FFPE was taken by GeoMx DSP (NanoString Technologies, Seattle, WA, USA) and uploaded in Qupath software (v. 0.5.0). For CD3 quantification nine ROIs with 90000 px^2 area/each were selected in each slide; three ROIs were selected exclusively in PanCk+/ S100B-PMEL17+ tumoral areas; three ROIs were selected exclusively in CD45+ immune infiltrated areas and three ROIs were selected in tumoral & immune infiltrated interactive areas (PanCk+/ S100B-PMEL17+ and CD45+ regions). For NeuN quantification nine ROIs with 90000 px^2 area/each were selected in each slide: three ROIs were located exclusively in PanCk+/ S100B-PMEL17+ tumoral areas; three ROIs exclusively in NeuN+ areas and three ROIs in tumoral & NeuN+ interacting areas (PanCk+/ S100B-PMEL17+ NeuN+ regions). Necrotic areas were excluded from all the FFPEs during ROI selection.Mean H-scores of CD3 and Neun in each ROI were automatically calculated using Qpath built in “Positive cell detection”(7). The optical signal threshold to classify the score into 4 bins was set to 10, 30, and 100. Thirteen slides from 13 different patients were evaluated and, in each slide, nine ROIs were selected following the described strategy for obtaining CD3 and NeuN H-scores. Statistical comparisons between groups were performed employing nonparametric one-tailed Mann-Whitney test according to data distribution with GraphPad Prism version 7.04 (GraphPad Software Inc., San Diego, CA).

**Immunohistochemistry (IHC) Analysis**

The expressions of the markers CD8 (clone SP57, Ventana), and S100B (clone XPS-21 Ventana) were assessed in56 brain-metastatic FFPEs. The expressions of the marker CD20 (clone L26, Ventana) was evaluated only on a subset of 54 FFPEs due to the lack of available specimens to be tested for the remaining patients. Additionally, the expression of the marker CD45 (clone RP2/18, Ventana) was evaluated in FFPE samples from both 7 PM and 13 MBM. Histopathological analyses were performed using the percentage of positively stained cells. Comparisons of IHC results from MBM vs non-MBM and PM vs MBM, respectively, were performed using Mann Whitney one tailed tests with GraphPad Prism version 7.04 (GraphPad Software Inc., San Diego, CA).

**Generation of tumor infiltrating lymphocytes (TILs) and Immunophenotyping**

Briefly, tumor tissue was dissected free of hemorrhagic and necrotic areas and cut into approximately 1-8 mm^3^ fragments. Some of the tumor fragments were used for generation of tumor digests by incubation in enzyme media [RPMI-1640 medium supplemented with 2 mM L-glutamine (Sigma Life Sciences, cat. no. G7513), 10 ug/mL gentamicin (GIBCO, cat. no. 15750060, 30 units/mL DNase and 1.0 mg/mL collagenase (both from Millipore Sigma, cat. no D4527 and C2674, respectively)] followed by mechanical dissociation. Tumor fragments and tumor digests (1x106 tumor digest cells) were then plated individually in 24-well plates and cultured in 2 mL of RPMI-1640 medium supplemented with 25 mM HEPES (Gibco, cat. no. 15630106), 100U/ml Penicillin/100μg/ml Strep (Gibco Cat no: 15140-122), 10 µg/mL gentamicin (Gibco, cat. 15750060) 10% human AB serum (Valley Biomedical USA, Cat no: HP1022HI, Lot no: 22Bo312) and 6000 IU/mL of interleukin (IL)-2 (Peprotech, Cranbury, NJ, USA) for 3-4 weeks. Medium was replenished first time on day 5, and after that about third times weekly; the wells were split in 1:2 fashion when fully confluent and cryopreserved until further use.

TIL clones were stained with surface antibodies specific for CD45-cFluor B548 (Cytek Biosciences, clone HI30, cat#RC-00113); CD3-cFluor R780 (Cytek Biosciences, clone SK7, cat# RC-00100); CD4-cFluor R840 (Cytek Biosciences, clone SK3, cat#R7-20165); CD8-BV570 (BioLegend, clone RPA-T8, cat#301038); CD56-SB780 (Cytek Biosciences, clone TULY56, cat#78-0566-42); CD16-cFluor B675 (Cytek Biosciences, clone 3G8, cat#RC-00523); TIGIT-BV421 (BD Biosciences, clone 7411820, cat#747844); CD279 (PD-1)-BB515 (BD Biosciences, clone EH12.1, cat#564494); CD223 (LAG3)-PerCP-eFluor 710 (Thermo Fisher Scientific, clone 3DS223H, cat#46-2239-42), CD159a (NKG2A)-BV711 (BD Biosciences, clone 131411, cat#747919) and ViaDye Red Fixable Viability Dye (Cytek Biosciences, R7-60008, cat#R7-60008). Statistical comparisons between groups were performed employing parametric unpaired t-test or nonparametric Mann-Whitney test according to data distribution with GraphPad Prism version 7.04 (GraphPad Software Inc., San Diego, CA).

**Overall survival analyses of patients**

Data available from n=51 specimens from BrMs (13 MBM, 18 BBM, 16 LBM and 4 RBM) were analyzed. For this exploratory analysis, we chose a single cutoff for each biomarker to divide patients into low and high biomarker groups. The same cutoff was applied across cancer types and calculation of OS (from diagnosis or from brain metastasis). Cutoff values were chosen based upon visual inspection of scatterplots of survival against the biomarker, while also attempting to choose values consistent with earlier standards. Due to known differences between brain metastases of different cancer types and evidence for interactions between cancer type and associations of biomarkers with OS in our own data, we analyzed associations between biomarker levels and OS separately for each type of primary cancer. All survival curves were calculated using the Kaplan-Meier method, and comparisons between biomarker groups were made using the log-rank test.

**Supplementary Materials and Methods References**

1. Ascierto ML, McMiller TL, Berger AE, Danilova L, Anders RA, Netto GJ, et al. The intratumoral balance between metabolic and immunologic gene expression is associated with anti–PD-1 response in patients with renal cell carcinoma. Cancer immunology research. 2016;4(9):726-33.

2. Kim D, Pertea G, Trapnell C, Pimentel H, Kelley R, Salzberg SL. TopHat2: accurate alignment of transcriptomes in the presence of insertions, deletions and gene fusions. Genome biology. 2013;14(4):1-13.

3. Pang Z, Chong J, Zhou G, de Lima Morais DA, Chang L, Barrette M, et al. MetaboAnalyst 5.0: narrowing the gap between raw spectra and functional insights. Nucleic acids research. 2021;49(W1):W388-W96.

4. Helmink BA, Reddy SM, Gao J, Zhang S, Basar R, Thakur R, et al. B cells and tertiary lymphoid structures promote immunotherapy response. Nature. 2020;577(7791):549-55.

5. Mendoza-Valderrey A, Alvarez M, De Maria A, Margolin K, Melero I, Ascierto ML. Next Generation Immuno-Oncology Strategies: Unleashing NK Cells Activity. Cells. 2022;11(19):3147.

6. Böttcher JP, Bonavita E, Chakravarty P, Blees H, Cabeza-Cabrerizo M, Sammicheli S, et al. NK cells stimulate recruitment of cDC1 into the tumor microenvironment promoting cancer immune control. Cell. 2018;172(5):1022-37. e14.

7. Bankhead P, Loughrey MB, Fernández JA, Dombrowski Y, McArt DG, Dunne PD, et al. QuPath: Open source software for digital pathology image analysis. Scientific reports. 2017;7(1):1-7.

**Supplementary Figure Legends**

**Supplementary Figure 1. Characterization of PM vs MBM tumor microenvironment.** **(A)** Representative data from positive and negative CD45 staining conducted on PM and MBM specimens are here shown with 20X magnification. Positive and negative CD45+ staining cells percentage was 70% and 0% for PM and MBM specimens, respectively. One-tailed Mann Whitney test was used to calculate P value. **(B)** Representative DSP images of a PM and a MBM samples. Morphology markers: S100B-PMEL17 (green), CD45 (pink) and DNA (blue). Volcano plot representations based on the expression of 56 proteins between 8 bulk AOIs-PM vs 8 bulk AOIS-MBM. Each dot of the graph corresponds to a protein. The fold difference in expression between the different groups is graphed on the x axis (logarithm of the base 2-fold changes). The p-value for each protein is graphed on the y axis (negative logarithm to the base 10). Dashed lines indicate the threshold of significant protein expression, defined ± Log2 FC ≥ 0.6 and -log10 (P) ≥ 1.3 after Lineal Mix Model analysis. Highlighted proteins on the volcano plots are the significantly enriched in MBM (right part, red) or PM (left part, blue) in bulk TME (tumor S100B-PMEL17+ and CD45+ compartments). Highlighted in black are other proteins with -log10 (P) ≥ 1.3. **(C)** Immune profiling of NK cells infiltration in PM and MBM TILs. Representative flow cytometry plots showing the gating strategy for CD56bright and CD56dim NK cells in a selected TIL PM clone (on top) and MBM TIL clone (on the bottom). The percentage of total CD3- CD56+, CD56bright, CD56dim evaluated by spectral flow cytometry between PM vs MBM are shown. One-tailed unpaired t-tests or one-tailed Mann Whitney tests were used to calculate P values.

**Supplementary Figure 2.** IHC analysis of paraffin-embedded tumor specimens (FFPE) derived from MBM vs non-MBM indicating pre-treated and treated patients at the moment of craniotomy and brain mets tissue collection.

**Supplementary Table Legends**

**Supplementary Table 1. Patient Characteristics**. *Total number of brain metastasis specimens evaluated= 57 derived from 53 unique patients; three BBM from the same pt and four LBM derived from two different pts (2 specimens/each), respectively, were here evaluated. Two FFPE specimens derived from primary melanoma (PM) with matching brain metastases were also analyzed in this study. One MBM and one PM fresh tissue specimens was here used for TIL generation and spectral flow cytometry analysis. Information regarding specimens used for the different assessments conducted in this study are provided in Table 2.

**Supplementary Table S2**: List of Illumina probes differentially expressed (Adjusted P ≤ 0.05) in MBM vs non-MBM patients.

**Supplementary Table S3**: List of the 56 selected immune related proteins assessed by DSP in MBM vs non-MBM patients.

**Supplementary Info S4**- Material and Methods
